# Semiquantitative Analysis for High-Speed Mapping Applications of Biological Samples Using LA-ICP-TOFMS

**DOI:** 10.1021/acs.analchem.3c01439

**Published:** 2023-04-26

**Authors:** Dino Metarapi, Andreas Schweikert, Ana Jerše, Martin Schaier, Johannes T. van Elteren, Gunda Koellensperger, Sarah Theiner, Martin Šala

**Affiliations:** † National Institute of Chemistry, Hajdrihova 19, 1000 Ljubljana, Slovenia; ‡ Institute of Analytical Chemistry, Faculty of Chemistry, University of Vienna, Waehringer Strasse 38, 1090 Vienna, Austria; § Institute of Inorganic Chemistry, Faculty of Chemistry, University of Vienna, Waehringer Strasse 42, 1090 Vienna, Austria; ∥ Vienna Doctoral School in Chemistry (DoSChem), University of Vienna, Waehringer Strasse 42, 1090 Vienna, Austria

## Abstract

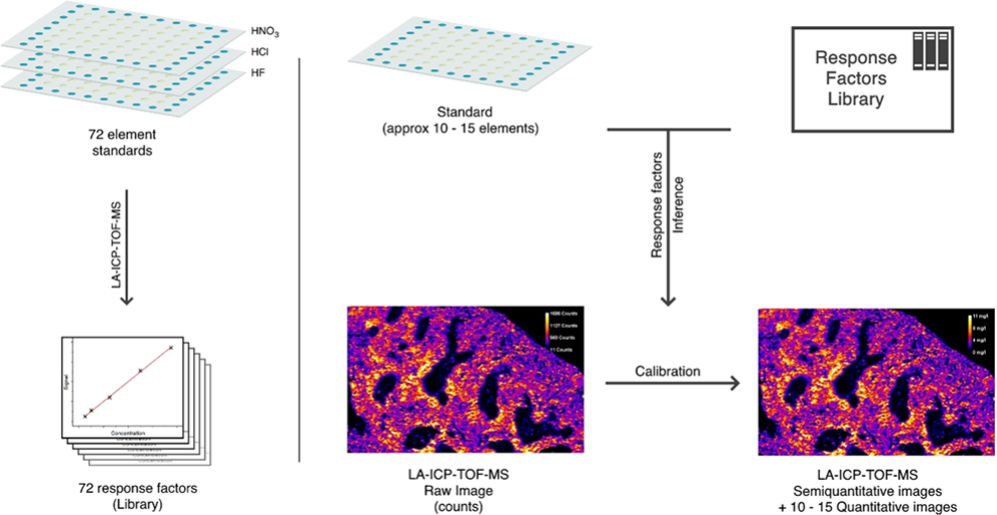

Laser ablation (LA) in combination with inductively coupled plasma time-of-flight mass spectrometry (ICP-TOFMS) enables monitoring of elements from the entire mass range for every pixel, regardless of the isotopes of interest for a certain application. This provides nontargeted multi-element (bio-)imaging capabilities and the unique possibility to screen for elements that were initially not expected in the sample. Quantification of a large range of elements is limited as the preparation of highly multiplexed calibration standards for bioimaging applications by LA-ICP-(TOF)MS is challenging. In this study, we have developed a workflow for semiquantitative analysis by LA-ICP-TOFMS based on multi-element gelatin micro-droplet standards. The presented approach is intended for the mapping of biological samples due to the requirement of matrix-matched standards for accurate quantification in LA-ICPMS, a prerequisite that is given by the use of gelatin-based standards. A library of response factors was constructed based on 72 elements for the semiquantitative calculations. The presented method was evaluated in two stages: (i) on gelatin samples with known elemental concentrations and (ii) on real-world samples that included prime examples of bioimaging (mouse spleen and tumor tissue). The developed semiquantification approach was based on 10 elements as calibration standards and provided the determination of 136 nuclides of 63 elements, with errors below 25%, and for half of the nuclides, below 10%. A web application for quantification and semiquantification of LA-ICP(-TOF)MS data was developed, and a detailed description is presented to easily allow others to use the presented method.

## Introduction

Inductively coupled plasma-mass spectrometry (ICP-MS) is an established analytical technique for elemental analysis at (ultra-)trace levels. For quantification, dedicated calibration strategies are required that are based on external calibration, standard addition, or isotope dilution approaches. The technique can be used for the determination of most elements in the periodic table, and although in theory all of them could be added into calibration solutions, this is generally not the practice. Only the elements of interest for a certain application are added into calibration solutions, and consequently, a limited number of elements can be quantified. To avoid losing information on concentration levels of other elements potentially present in the samples, semiquantitative methods have been developed for solution-based ICP-MS analysis. In semiquantitative analysis, the instrument performs a fast scan over the entire mass range and provides information on the elemental composition of the sample. Based on a predetermined response factor curve, the concentrations of all elements are assessed.^1−5^ The accuracy of semiquantitative analysis can be improved by adjusting the response factor curve using a calibration curve containing only several elements in two or even only one concentration level(s).^2,6^ Semiquantitative analysis is well established for solution-based ICP-MS and has been evaluated for different sample types. In general, good agreement was found for most elements between certified values and values that were determined in a quantitative or semiquantitative way.^2,3,6,7^

Coupling laser ablation (LA) with ICP-MS enables multi-element analysis of solid samples without laborious sample preparation, and the method has been applied to a variety of sample types ranging from geological to biological samples.^8,9^ Quantification in LA-ICPMS, in general, is challenging as it requires standards that match the sample matrix as closely as possible to mimic the processes during the ablation and ionization steps of the analysis. Since these are rarely available, especially for biological samples, different strategies have been developed for calibration.^10^ Quantification approaches are, for example, based on homogenization of different tissue types such as brain^11^ or liver^12^ and standard addition of elements of interest, followed by sectioning the standards to the same thickness as the sample. Alternative strategies rely on the preparation of external standards based on gelatin as a matrix, which is considered to mimic the properties of biological samples.^13^ For quantification by LA-ICPMS, gelatin-based calibration standards in the form of sections,^14,15^ microarrays,^16^ bioprinted standards,^17^ and droplets^15,18^ have been proposed. The latter ones were further developed as micro-droplet standards^19,20^ to increase throughput in total ablation approaches by decreasing their size through automated and precise deposition by a micro-spotting device.^20,21^ Moreover, aspiration of a standard solution (in-cell or in-torch) during laser sampling and applications of a polymer-based thin film standard on/under a biological sample combined with total consumption (i.e., ablation of the entire depth) of the assembly have been reported.^22−25^ Different approaches for semiquantitative analysis by LA-ICPMS have been described in the literature. The most commonly used primary standards in LA-ICPMS analysis of geological matrices are the NIST SRM 610/611 and 612/613 glasses, which have a different matrix compared to any naturally occurring geological material. In this case, internal standardization is critical, as it allows semiquantitative calibration using a standard that is not matrix-matched with the samples.^26^ Internal standardization corrects, to some extent, for matrix suppression/enhancement effects and signal drifts in the ICP-MS. Alternatively to the use of a single internal standard element, signal sum normalization can be applied by normalizing the total element concentrations to 100% abundance, providing semiquantitative analysis.^26,27^

In most cases, quadrupole-based MS (Q MS) systems are used in LA-ICPMS setups, enabling the sequential measurement of the selected mass-to-charge ratios (*m*/*z*). The development of low-dispersion LA setups in recent years has shortened the single pulse responses (SPR) of each laser shot to <1 ms,^28−31^ and hence only one, or in the best case, a few nuclides can be measured by ICP-QMS detection. Therefore, the combination of low-dispersion LA systems and ICP-time-of-flight MS (ICP-TOFMS) instruments is advantageous for fast transient signals as all *m*/*z* values are measured quasi-simultaneously.^32^ In this case, the entire mass range is monitored for every laser shot, regardless of the isotopes of interest for a certain application. As a result, this gives the opportunity to scan for elements one may not initially expect in the samples. However, without standards, only qualitative data can be obtained. The development of a semiquantitative approach for LA-ICP-TOFMS would therefore be a useful tool to provide an overview on the concentrations of a range of elements present in a biological sample.

In this study, we have developed a semiquantitative LA-ICP-TOFMS approach for biological samples and a corresponding web-based application that works on the same principles as the semiquantitative analysis in solution-based ICP-MS analysis. Multi-element quantification was performed based on different sets of multi-element gelatin microdroplets as calibration standards that have proved to be good matrix-matched standards for biological samples.^21^ Based on the obtained data, a library of response factors was constructed, and a software was created that provides the calculations of elemental concentrations from the user’s LA-ICP-TOFMS data sets in a quantitative and semiquantitative manner. As proof of principle, we have focused on the multiplexed LA-ICP-TOFMS analysis of biological samples and showcased the developed semiquantitative approach on thin sections of mouse spleen and tumor tissue.

## Experimental Section

### Chemicals and Reagents

Ultrapure water (18.2 MΩ cm, ELGA water purification system, Purelab Ultra MK 2, U.K.) and nitric acid (>69%, ROTIPURAN Supra, Carl Roth, Karlsruhe, Germany) were used for all dilutions of the standard solutions. A multi-element stock solution was purchased from LabKings (Hilversum, the Netherlands) and (ICP)-grade single-element standards were purchased from either Merck (CertiPUR, Germany) or LabKings (Hilversum, the Netherlands). The detailed list of elements is available in Table S1. Gelatin was obtained from Sigma-Aldrich (Vienna, Austria). Solution preparations and measurements were carried out in clean room classes ISO 8 and ISO 7, respectively.

### Preparation of Gelatin-Based Micro-Droplet Standards

Gelatin-based micro-droplet standards were prepared according to a previously described procedure.^20^ Three different types of standards were prepared: (1) a multi-element stock solution containing 48 elements (Multi48-standards) in HNO_3_, (2) single-element standard stock solutions in HF and HNO_3_ were pooled together (HF/HNO_3_-standards), and (3) single-element standard stock solutions in HCl (HCl-standards) were pooled together. The multi-element standard solutions Multi48-standards and HF/HNO_3_-standards were serially diluted in 1% (v/v) nitric acid, and the HCl-standards were serially diluted in ultrapure water. Each type of gelatin standard set was prepared in triplicate. The elements present in the different gelatin standard batches with their corresponding absolute amounts can be seen in Tables S2–S4. In addition, two sets of standards were prepared from a multi-element stock solution containing 26 elements. One set was used as the calibration standard, and the second set was treated as the sample. These standards were serially diluted in 1% (v/v) nitric acid. The elements present in these standards with their corresponding absolute amounts can be seen in Tables S5 and S6.

The resulting multi-element solutions were spotted via a cellenONE X1 micro-spotter (Cellenion, Lyon, France) onto glass slides. The volume of the droplets was assessed optically by the software of the instrument, with droplet volumes of 370 ± 10 pL and sizes of around 150–200 μm in diameter on the glass slide after drying. The microdroplets were spaced with distances of around 150 μm. The slides were stored at room temperature until LA-ICP-TOFMS analysis.

### Mouse Tissue Sections

For in vivo experiments, 1 × 10^6^ HCT116 cells were injected subcutaneously, in serum-free Roswell Park Memorial Institute (RPMI)-medium (R6504, Sigma-Aldrich, St. Louis, MO), into the right flank of 11-week-old male CB-17/SCID mice. The animals were kept in a pathogen-free environment and handled in a laminar airflow cabinet. Animal experiments were performed according to the regulations of the Ethics Committee for the Care and Use of Laboratory Animals at the Medical University of Vienna (proposal number BMWF-66.009/0140-II/3b/2011), the U.S. Public Health Service Policy on Human Care and Use of Laboratory Animals, as well as the United Kingdom Coordinating Committee on Cancer Prevention Research’s Guidelines for the Welfare of Animals in Experimental Neoplasia. The animals were controlled for symptoms of distress daily, and tumor size was assessed regularly by caliper measurement. On day 17, the mice were sacrificed. Tumor and spleen were formalin-fixed in 4% formaldehyde for 24 h (Carl Roth, # P087.3) and paraffin-embedded using a KOS machine (Milestone Medical, Sorisole, Italy). The embedded samples were cut in sections of 5 μm thickness and mounted onto glass slides. Tissue sections were deparaffinized and labeled with a set of 17 metal-conjugated antibodies following standard protocols according to a previously published study.^33^

### LA-ICP-TOFMS Measurement

An Iridia 193 nm excimer laser ablation system (Teledyne Photon Machines, Bozeman, MT) was coupled to an *icp*TOF 2R (TOFWERK AG, Thun, Switzerland) TOF-based ICP-MS instrument. The LA system was equipped with a low-dispersion ablation cell^31^ within the cobalt ablation chamber and connected to the ICP-TOFMS system via the aerosol rapid introduction system (ARIS). Through the low-dispersion mixing bulb of the ARIS, an Ar makeup gas flow (∼0.90–1.0 L min^–1^) was introduced into the optimized He carrier gas flow (0.60 L min^–1^) before entering the plasma. The LA and ICP-TOFMS settings were optimized on a daily basis while ablating NIST SRM612 glass-certified reference material (National Institute for Standards and Technology, Gaithersburg, MD). Optimization was based on high intensities for ^24^Mg^+^, ^59^Co^+^, ^115^In^+^, and ^238^U^+^, low oxide formation was based on the ^238^U^16^O^+^/^238^U^+^ ratio (<2%), and low elemental fractionation was based on the ^238^U^+^/^232^Th^+^ ratio (∼1). Laser ablation sampling was performed in fixed dosage mode 2 at a repetition rate of 200 Hz and using a 5 μm × 5 μm square spot. The line scans overlapped one another by 2.5 μm. Selective ablation of the gelatin microdroplets and tissue sections was achieved by selecting an energy density below the ablation threshold of glass and above the ablation threshold of gelatin.^34^ Gelatin microdroplets and tissue sections were removed quantitatively using a fluence of 0.60 J cm^–2^.^35^

The *icp*TOF 2R ICP-TOFMS instrument has a specified mass resolution (*R* = *m*/Δ*m*) of 6000 (full width at half-maximum definition). The standard operation mode was used, which balances mass resolving power, sensitivity, and ion transmission across the entire measured mass range and which allows the analysis of ions from *m*/*z* = 14–256. The integration and read-out rate match the LA repetition rate. The instrument was equipped with a torch injector of 2.5 mm inner diameter and nickel sample and skimmer cones with a skimmer cone insert of 2.8 mm in diameter. A radio frequency power of 1440 W, an auxiliary Ar gas flow rate of 0.80 L min^–1^, and a plasma Ar gas flow rate of 14 L min^–1^ were used. For all measurements, the collision cell technology (CCT) mode was used, where the collision cell was pressurized with a mixture of H_2_/He gas (93% He (v/v), 7% H_2_ (v/v)), with an optimized flow rate of 4.2 mL min^–1^. Instrumental parameters for ICP-TOFMS measurements are summarized in Table S7.

### Data Acquisition and Processing

Data was recorded using TofPilot v.2.11.6.0.190ff674 (TOFWERK AG, Thun, Switzerland). The LA-ICP-TOFMS data were saved in the open-source hierarchical data format (HDF5, www.hdfgroup.org). Post-acquisition data processing was performed with Tofware v3.2.2.1, which is a TOFWERK data analysis package and used as an add-on on IgorPro (Wavemetrics Inc., Oregon). The data processing comprised the following steps: (1) drift correction of the mass peak position in the spectra over time via time-dependent mass calibration (2) determining the peak shape and (3) fitting and subtracting the mass spectral baseline. Data was further processed with HDIP version 1.6.6.d44415e5 (Teledyne Photon Machines, Bozeman, MT). An integrated script was used to automatically process the files generated by Tofware and to generate two-dimensional (2D) elemental distribution maps. For calibration, signal responses for each mass channel monitored during ablation of a single spiked droplet were integrated using HDIP. The integrated signal intensities and the absolute masses of the respective elements within the gelatin micro-droplet standards were used to set up calibration curves.

Data processing for the semiquantitative calibration and custom-developed semiquantitative calibration script was packaged in an online app by MatLab R2020a (MathWorks, Natick, MA). Image processing and visualization were performed in ImageJ 1.53.

## Results and Discussion

The concept of semiquantitative calibration takes into account that predetermined response factors for each nuclide, defined as intensity per unit concentration, are used for quantification of real samples. Therefore, in semiquantitative approaches for ICP-MS analysis, a library of response factors for the highest number of available nuclides has to be constructed to be able to predict their concentrations in real-world samples. The set of nuclides used for this study was composed of a series of multi-element and individual calibration standards (summarized in Table S1) for the library construction. For this purpose, different batches of gelatin micro-droplet standards had to be prepared due to the following reasons: (i) the compatibility of the element standard stock solutions (e.g., silver would precipitate if mixed with HCl, which is contained in other element standard solutions); (ii) it is very impractical to mix several single-element standards in one go; and (iii) with increasing number of elements in gelatin as matrix, the gelatin becomes brittle, precipitation can occur and it becomes difficult to handle, especially at higher elemental concentrations. We have already shown in a previous study^21^ that microdroplets based on gelatin are valid matrix-matched standards for biological samples (tissue sections and cells). Therefore, the presented semiquantitative approach is intended for the LA-ICPMS mapping of biological samples.

There is a multitude of factors that affect the sensitivity of an MS toward different nuclides. In this study, an algorithm was chosen that does not make any underlying assumptions about the LA-ICP-TOFMS processes. The LA-ICP-TOFMS was therefore treated as a “black box.” Based on the different gelatin standard batches, a library of response factors was constructed for the semiquantitative calculations. The library consisted of measured sensitivities toward the masses of elements contained within the gelatin microdroplets (in total 72 elements). A linear regression analysis was performed for all of the elements (five concentration levels, three replicates each), whereupon all linear fits with an *R*^2^ < 0.95 were excluded from consideration. The *R*^2^ values for the nuclides that were used to create the library are summarized in Table S8. The following 10 elements that were below the threshold of *R*^2^ < 0.95 were not included in the library: ^7^Li, ^9^Be, ^11^B, ^23^Na, ^24^Mg, ^27^Al, ^31^P, ^39^K, ^43^Ca, and ^45^Sc.

From the known elemental concentrations of the standards, the slopes (*a*) and intercepts (*b*) were stored in single-column arrays (eq 1).
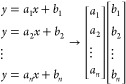
1After this, gelatin microdroplets with known elemental concentrations that were treated as test samples were also analyzed by LA-ICP-TOFMS. From these test samples, another set of slopes (*p*) and intercepts (*q*) were obtained (eq 2).
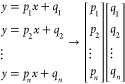
2The accuracy of the developed semiquantitative approach was evaluated in two stages. First, an independent set of microdroplets was treated as a sample, and the SQ results were compared to the actual concentration. This set of microdroplets was used to simplify the evaluation process, as the exact elemental concentrations were known. The second evaluation of the semiquantification approach was based on tissue samples and provided the real-world scenario, with either (i) the “real” quantitative calibration by constructing all of the calibration curves or (ii) by including some of the elements for quantitative calibration and predicting all others with the developed semiquantitative approach. With two full sets of slopes and intercepts derived for both the library and samples, it was possible to test the semiquantitative calibration strategy by comparing the quantitative calibration and semiquantitative one.

**Figure 1:**
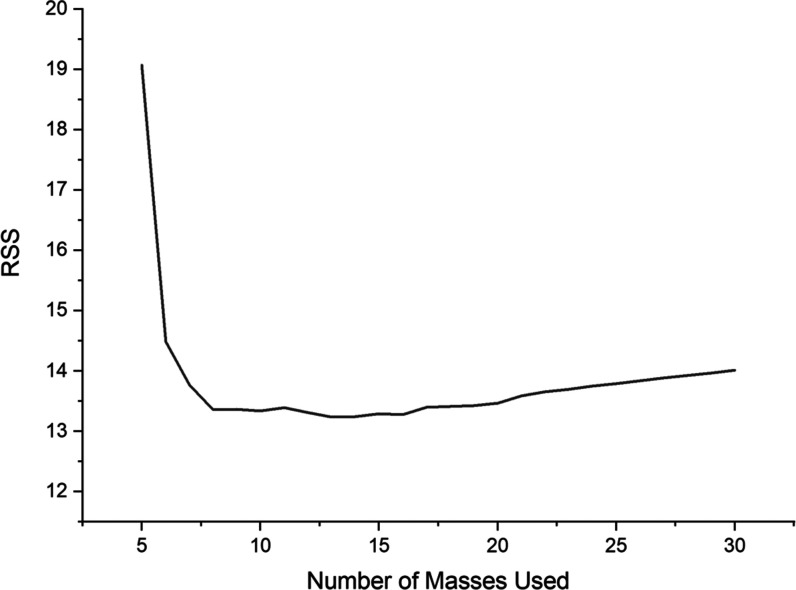
Sum of the squares of residuals in the plot shows the results obtained from 1 million iteration bootstrapping procedures for different numbers of elements used for the semiquantitative approach.

In the first test, a bootstrapping procedure was devised, which used the ratio between library slopes (*a*) and random sampling of sample slopes (*p*) in order to estimate the best selection of masses for the semiquantitative calibration approach. Selections ranging from 5 to 30 masses were tested with 1 million iterations. The selected masses were then used for the semiquantitative approach to predict the other nuclide concentrations. In short, using three different interpolation algorithms (modified Akima, pchip, and spline), the unknown ratios (*u*_1_, *u*_2_, ···, *u_n_*) were inferred from the slope ratios (*r*_1_, *r*_2_, ···, *r_n_*) obtained by dividing the slopes from the library (*a*_1_, *a*_2_, ···, *a_n_*) and the randomly selected sample slopes (*p*_1_, *p*_2_, ···, *p_n_*) using eq 3 (where *r*_(*n*,*i*)_ are the inferred ratios).
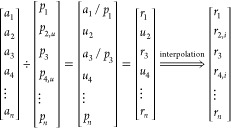
3From these, the library slopes were used again to infer the unknown slopes (eq 4), where *p*_(*n*,*i*)_ stand for the inferred slopes.
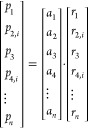
4Where available, the slopes obtained by linear regression were used. In order to obtain the inferred intercepts (*q*_(1,*i*)_, *q*_(2,*i*)_, ···, *q*_(*n*,*i*)_), the library intercepts (*b*_1_, *b*_2_, ···, *b_n_*) were scaled by a factor equal to the ratios of library and sample slopes (eq 5).
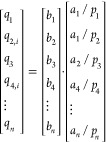
5As with the sample slopes, where available, the linear regression-obtained intercepts (*q*_1_, *q*_2_, ···, *q_n_*) were used. Using the obtained slopes and intercepts, the elemental concentrations from each ablated micro-droplet were calculated. The procedure was repeated 1 million times for 5 elements, 6 elements up to 30 elements chosen for the standards in the SQ approach. A detailed overview of the workflow of the bootstrapping procedure can be found in Figure S1. For each iteration of these 1 million in the bootstrap procedure, the sum of the squares of residuals (RSS) from the deviation of inferred concentrations from the true concentration was calculated; the outcome is presented in Figure 1. It can be seen that the error dropped with the number of standards used for the SQ approach, which is to be expected. An important observation was that the error did not improve significantly when more than 10–15 elements were used as standards for SQ calibration. Of course, the best scenario would be to use all of the standards to get truly quantitative results, but as described already, it is practically impossible. By sorting the results from the lowest to the highest RSS, an optimal selection of masses could be performed. It is worth noting, however, that the number of iterations of 1 million was chosen for computational reasons, as the true number of nonrepeating combinations would range from 2.48 × 10^4^ to 1.40 × 10^15^, depending on the number of masses used for the semiquantitative calibration. The selection of masses used for semiquantitative calibration with the lowest RSS should therefore not be taken as an absolute optimum, which does exist in principle, although the computational requirements to obtain it are prohibitive.

The analysis of the bootstrapping procedure, namely, the nuclides used as standards in the results of the SQ calibrations, where the RSS was lowest, yielded the 10 nuclides that were represented in the most combinations of experiments (in silico). It has to be considered that the 10 nuclides were chosen randomly (1 million times), and the differences in RSS within the combinations of nuclides yielding the lowest RSSs were relatively low. Therefore, the nuclides with the highest occurrence were chosen and are presented in Table S1 (in the order from the nuclides that are most commonly represented in lowest RSS yielding SQ to lower ones). Of course, these elements will be determined quantitively, all of the others semiquantitively. Therefore, we recommend adding the most important nuclides that need to be determined quantitively to the mix used as calibration standards. In the end, one would end up with only a small number of elements in addition to the usual analytes. This is reasonably easy to handle, even at higher concentration ranges.

**Figure 2:**
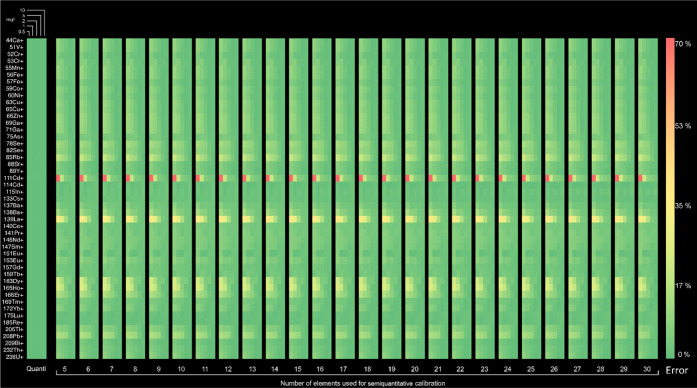
Heatmaps of the percentage deviations from the true value for quantitative and semiquantitative calibrations. Each semiquantitative calibration heatmap was made using the selection of elements resulting in the lowest RSS. Every square represents an average of three replicates.

The heatmap in Figure 2 represents the deviation of the results from the semiquantitative approach as compared to the quantitative approach. The concentration results were subtracted from the true concentration values, and the deviation (in percentages) is shown, with green representing the least deviation and red the highest. It can be observed that even for a relatively low number of masses used for the semiquantitative calibration, the percentage deviation still remained below 25% and approached that of quantitative calibrations, especially when using more than 10 masses. On the *x* scale, the number of elements used as standards for the SQ prediction are shown in bars (each bar is used for a certain number of elements, divided into five columns) representing the increasing concentrations, while the elements of interest are listed in vertical rows.

**Figure 3:**
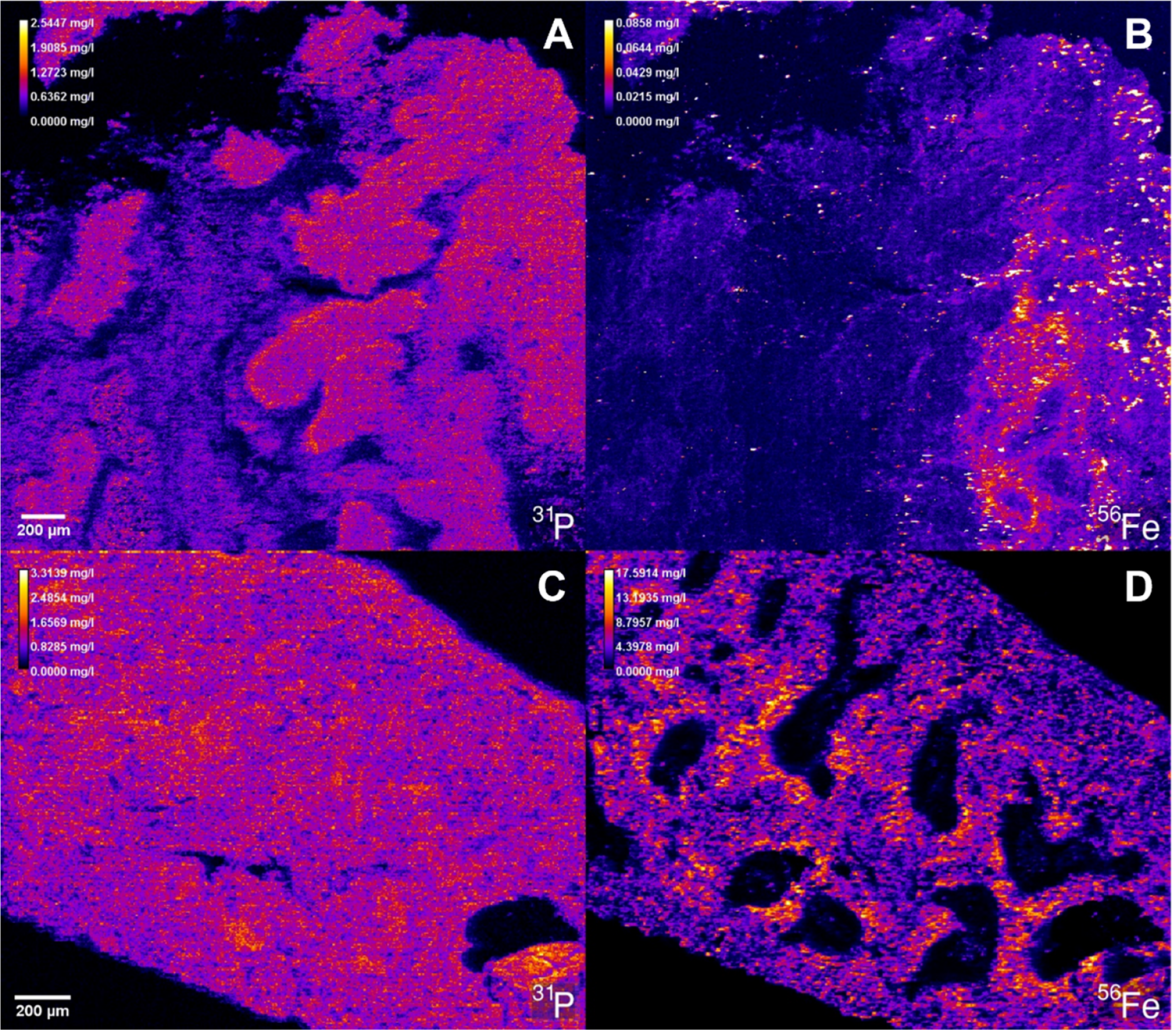
Concentration maps of (A) ^31^P^+^ and (B) ^56^Fe^+^ in a mouse tumor section, and of (C) ^31^P^+^ and (D) ^56^Fe^+^ in a mouse spleen section, determined by the semiquantitative method and LA-ICP-TOFMS analysis.

The methodology was further tested on two biological samples: (i) a thin section of a murine tumor and (ii) a thin section of a mouse spleen, both stained with 17 metal-tagged antibodies to visualize different cell types, functions, and states.^33^ Examples of elemental maps displaying endogenous elements in mouse tumor and spleen are depicted in Figure 3, whereas the phosphorus and iron concentrations were determined using the semiquantitative approach. The phosphorus signal can be used to visualize the tissue structure in the tumor and the spleen. Especially in the tumor microenvironment, reduced phosphorus concentrations can be indicative of necrotic areas due to DNA degradation compared to living tissue. For iron, high concentration levels in certain regions point toward the presence of blood vessels. In the spleen, the red pulp exhibited significantly higher iron values than the white pulp, which is in accordance with previous LA-ICPMS results.^33^

**Figure 4:**
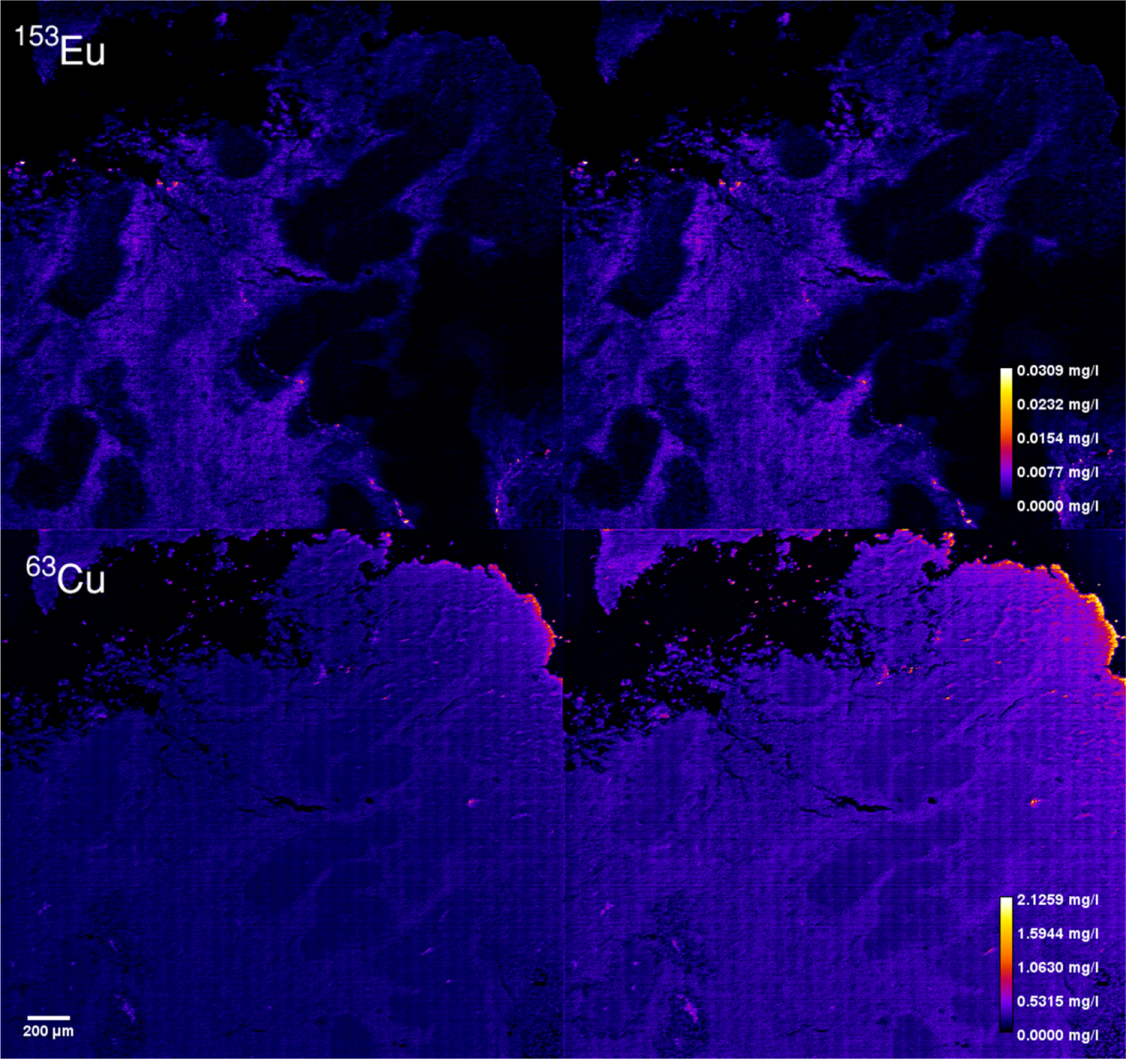
Elemental maps of a murine tumor section representing the quantitative maps of ^153^Eu and ^63^Cu. On the left part, the elemental maps based on the quantitative approach, and on the right part, the elemental maps based on the semiquantitative approach are shown.

For the evaluation of the developed method on biological samples, 48 element-containing gelatin micro-droplet standards were used for quantitative and semiquantitative calibration. In a first step, the calibration was performed with all of the 48 elements in a conventional way by external calibration and by calculating the elemental concentrations from the respective calibration curves. Then, a few iterations of the semiquantitative approach were performed to be able to estimate the error for all of the elements used as standards. The elements used for calibration in the semiquantitative approach were effectively quantitatively assessed; therefore, the procedure had to be repeated several times to retrieve the errors for all of the elements (using different combinations of elements used as standards). After each iteration, the values obtained in the SQ approach were subtracted from the values obtained from the quantitative approach, resulting in the errors of the SQ approach (summarized in Table 1). In short, approximately more than 80% of the nuclides could be measured with a precision better than 30%, and approximately one-third of the nuclides showed errors lower than 5%. The elements with errors that were well above the 30% (or below −30%) margin could be attributed to elements that suffer from isobaric interferences, intrinsic difficulties in measuring with TOF instruments (e.g., low mass range), or from interferences resulting from the sample matrix itself. Furthermore, it also shows that the quantitative approach is not free of errors (see the first bar in Figure 2, typically below 1%), depicting the differences between the concentrations determined by the quantitative approach and the actual concentrations of the prepared micro-droplet standards. The nuclides from the set included in the standards in the SQ approach were chosen randomly, and the errors reported are the average of five measurements (the nuclides used as standards can be found in Table S9). If the selection of nuclides is chosen more systematically, the errors can be also further decreased. The semiquantitative LA-ICP-TOFMS method was applied to a murine tumor section and compared to quantification by external calibration (Figure 4). Two of the nuclide maps measured are represented in Figure 4. For ^153^Eu (metal-conjugated antibody representing connective tissue) and ^63^Cu with errors in prediction of 0.9% and 40.9% respectively, a resemblance can be observed in the case of europium and a bit lower contrast in the copper map. It has to be noted that it is hard to represent the differences as the contrast in maps masks the actual differences. It would be ideal to compare the SQ measurements to the actual elemental concentrations in the biological sample, but this is not possible in this experimental setup.

**Table 1:** Average Errors Obtained for Five Random Sets of Elements Chosen for the Semiquantification Approach^a^

	tumor	spleen		tumor	spleen
nuclide	error (%)	error (%)	nuclide	error (%)	error (%)
^27^Al	–88.4	–88.4	^137^Ba	–22.7	–7.6
^44^Ca	–15.9	–11.5	^138^Ba	–31.9	0.9
^51^V	–8.8	–6.8	^139^La	–2.9	–2.2
^52^Cr	–27.4	–22.3	^141^Pr	0.9	0.4
^55^Mn	–24.0	–18.1	^146^Nd	–2.8	–2.7
^57^Fe	–17.4	–14.6	^147^Sm	2.6	2.6
^59^Co	–27.1	–27.8	^153^Eu	–0.9	–1.2
^60^Ni	–24.0	–26.3	^157^Gd	1.0	0.6
^63^Cu	–20.4	–26.0	^159^Tb	1.6	2.0
^64^Zn	37.0	26.1	^163^Dy	3.9	4.6
^65^Cu	–22.7	–29.3	^165^Ho	3.6	4.7
^66^Zn	45.6	60.5	^166^Er	4.5	5.6
^69^Ga	33.6	21.8	^169^Tm	4.9	6.3
^75^As	9.9	7.6	^172^Yb	10.0	8.1
^78^Se	–18.8	–18.4	^175^Lu	5.3	7.1
^85^Rb	107.9	105.5	^185^Re	2.5	4.8
^88^Sr	–7.9	–1.5	^205^Tl	64.3	67.2
^89^Y	–4.7	–1.5	^208^Pb	46.9	49.4
^114^Cd	–41.0	10.9	^209^Bi	33.3	35.5
^115^In	–34.9	–22.9	^232^Th	13.4	13.9
^133^Cs	223.8	230.1	^238^U	29.4	29.4

a Each individual error is the difference between the quantification and semiquantification approaches (average of *n* = 5 repetitions).

## Conclusions

A semiquantitative calibration approach was developed for LA-ICP-TOFMS bioimaging, and it showed that only a standard set with a limited number of nuclides was required for the prediction of numerous nuclides with a deviation below 25%, and mostly below 10%. This eases the preparation of gelatin standards for bioimaging applications, making (i) higher concentrations possible without the gelatin-related problems and (ii) providing calibration of nuclides that are not miscible in standards. The main advantage of the approach lies in the “measure all nuclides all the time” TOF approach. One can also have an “all nuclides (semi-)quantified all the time” approach. This also makes the data reprocessing and semiquantification of certain elements of interest that were not calibrated at the time possible. An app was written to easily allow others to use the developed semiquantitative method. A link to the online app as well as a detailed description of how to use the app can be found in the Supplementary Information (see Figures S1–S8).

## Supporting Information Available

Overview of the elements in the different sets of gelatin standards used for muti-element quantification and for the construction of the library for the semiquantitative approach (Table S1); the amount of each analyte added to the gelatin-based micro-droplet standards of the “Multi48” standard set (Table S2); the amount of each analyte added to the gelatin-based micro-droplet standards of the “HF/HNO_3_” standard set (Table S3); the amount of each analyte added to the gelatin-based micro-droplet standards of the “HCl” standard set (Table S4); the amount of each analyte added to the gelatin-based micro-droplet standards of the “Multi26_std” standard set (Table S5); the amount of each analyte added to the gelatin-based micro-droplet standards of the “Multi26_sample” standard set (Table S6); instrumental parameters for ICP-TOFMS measurements (Table S7); the *R*^2^ values for the nuclides that were used to create the library (Table S8); the nuclides chosen from the set included in standards in the semiquantification approach (Table S9); overview on the workflow of the bootstrapping procedure used for the semiquantitative calculations (Figure S1); and detailed application instructions (Figures S2–S9) (PDF)
