# Supplementary material for: Semiquantitative Analysis for High-Speed Mapping Applications of Biological Samples Using LA-ICP-TOFMS
Source: Anal Chem. 2023 Apr 26;95(19):7804–12. doi: 10.1021/acs.analchem.3c01439 (PMC10193359; doi:10.1021/acs.analchem.3c01439)
Supplement: Supplementary file 1 — ac3c01439_si_001.pdf [file ac3c01439_si_001.pdf]

Supplementary Information to

Semiquantitative analysis for high-speed mapping applications of biological samples using LA-ICP-TOFMS

Dino Metarapi[1], Andreas Schweikert[2,3], Ana Jerše[1], Martin Schaier[2,4], Johannes T. van Elteren[1], Gunda Koellensperger[2], Sarah Theiner[2*], Martin Šala[1*]

[1] National Institute of Chemistry, Hajdrihova 19, 1000, Ljubljana, Slovenia

[2] Institute of Analytical Chemistry, Faculty of Chemistry, University of Vienna, Waehringer Strasse 38, 1090 Vienna, Austria

[3] Institute of Inorganic Chemistry, Faculty of Chemistry, University of Vienna, Waehringer Strasse 42, 1090 Vienna, Austria

[4] Vienna Doctoral School in Chemistry (DoSChem), University of Vienna, Waehringer Strasse 42, 1090 Vienna, Austria

Corresponding authors:

sarah.theiner@univie.ac.at

martin.sala@ki.si

Outline

Table S1. Overview of the elements in the different sets of gelatin standards used for muti-element quantification by LA-ICP-TOFMS and for the construction of the library for the semiquantitative approach. Selection of recommended nuclides for the preparation of standards for the semiquantitative analysis.

Table S2. The amount of each analyte added to the respective gelatin-based micro-droplet standards for the three technical replicates of the 'Multi48' standard set.

Table S3. The amount of each analyte added to the respective gelatin-based micro-droplet standards for the three technical replicates of the 'HF/HNO_3' standard set.

Table S4. The amount of each analyte added to the respective gelatin-based micro-droplet standards for the three technical replicates of the 'HCl' standard set.

Table S5. The amount of each analyte added to the respective gelatin-based micro-droplet standards of the three technical replicates of the 'Multi26_std' standard set.

Table S6. The amount of each analyte added to the respective gelatin-based micro-droplet standards of the three technical replicates of the 'Multi26_sample' standard set.

Table S7. Instrumental parameters for ICP-TOFMS measurements.

Table S8. The nuclides chosen from the set included in standards in the semiquantification approach.

Figure S1. Overview on the workflow of the bootstrapping procedure used for the semiquantitative calculations.

Figure S2 – S9. Detailed application instructions.

Table S1. Overview of the elements in the different sets of gelatin standards for multi-element quantification by LA-ICP-TOFMS used for the construction of the library for the semiquantitative approach. The last column presents the selection of recommended nuclides for the preparation of standards for the semiquantitative analysis yielding the lowest errors in concentration prediction by the semiquantitative approach.

**Gelatin 1 - 48 multi-element STD in HNO_3**	**Gelatin 2 – 7 elements STDs in HCl**	**Gelatin 3 – 17 element STDs in HF/HNO_3**	**Recommended nuclides for selection of SQ standards**
element	*element*	*element*	*nuclide*
Li, Be, B	Mo	Si	^{51}V
Na, Mg	Ru	S	^{66}Zn
Al	Sn	Ti	^{78}Se
P	Os	Ge	^{85}Rb
K, Ca	Ir	Zr	^{88}Sr
Sc, V, Cr	Pt	Nb	^{89}Y
Mn	Au	Rh	^{133}Cs
Fe		Pd	^{137}Ba
Co, Ni		Ag	^{139}La
Cu, Zn		Sb	^{147}Sm
Ga		Te	^{151}Eu
As		Hf	^{153}Eu
Se		Ta	^{165}Ho
Rb, Sr		W	^{169}Tm
Y		Hg	^{172}Yb
Cd			
In			
Cs, Ba			
La, Ce			
Pr, Nd			
Sm, Eu			
Gd			
Tb, Dy			
Ho, Er			
Tm, Yb			
Lu			
Re			
Tl, Pb			
Bi			
Th, U			

Table S2. The amount of each analyte added to the respective gelatin-based micro-droplet standards for the three technical replicates of the 'Multi48' standard set. Multi48 symbolizes the analytes from a 48 element standard solution containing Li, Be, B, Na, Mg, Al, P, K, Ca, Sc, V, Cr, Mn, Fe, Co, Ni, Cu, Zn, Ga, As, Se, Rb, Sr, Y, Cd, In, Cs, Ba, La, Ce, Pr, Nd, Sm, Eu, Gd, Tb, Dy, Ho, Er, Tm, Yb, Lu, Re, Tl, Pb, Bi, Th, U.

Amount [fg]	Blank	Std 1	Std 2	Std 3	Std 4	Std 5
Multi 48	0	200	400	800	2000	4000

Table S3. The amount of each analyte added to the respective gelatin-based micro-droplet standards for the three technical replicates of the 'HF/HNO_3' standard set.

Amount [fg]	Blank	Std 1	Std 2	Std 3	Std 4	Std 5
Si	0	200	400	800	2000	4000
S	0	200	400	800	2000	4000
Ti	0	200	400	800	2000	4000
Ge	0	200	400	800	2000	4000
Zr	0	200	400	800	2000	4000
Nb	0	200	400	800	2000	4000
Rh	0	200	400	800	2000	4000
Pd	0	200	400	800	2000	4000
Ag	0	200	400	800	2000	4000
Sb	0	200	400	800	2000	4000
Te	0	200	400	800	2000	4000
Hf	0	200	400	800	2000	4000
Ta	0	200	400	800	2000	4000
W	0	200	400	800	2000	4000
Hg	0	200	400	800	2000	4000

Table S4. The amount of each analyte added to the respective gelatin-based micro-droplet standards for the three technical replicates of the 'HCl' standard set.

Amount [fg]	Blank	Std 1	Std 2	Std 3	Std 4	Std 5
Mo	0	200	400	800	2000	4000
Ru	0	200	400	800	2000	4000
Sn	0	200	400	800	2000	4000
Os	0	200	400	800	2000	4000
Ir	0	200	400	800	2000	4000
Pt	0	200	400	800	2000	4000
Au	0	200	400	800	2000	4000

Table S5. The amount of each analyte added to the respective gelatin-based micro-droplet standards of the three technical replicates of the 'Multi26_std' standard set. Multi26 symbolizes the analytes from a 26-element standard solution containing Be, B, Na, Mg, Al, Si, K, Ca, Ti, V, Cr, Mn, Fe, Co, Ni, Cu, Zn, As, Se, Mo, Ag, Cd, Sb, Ba, Tl, and Pb.

Amount [fg]	Blank	0.5	1	2	5	10
Mix	0	200	400	800	2000	4000
Si	0	100	200	400	1000	2000
K	0	2000	4000	8000	20000	40000

Table S6. The amount of each analyte added to the respective gelatin-based micro-droplet standards of the three technical replicates of the 'Multi26_sample' standard set. Multi26 symbolizes the analytes from a 26-element standard solution containing Be, B, Na, Mg, Al, Si, K, Ca, Ti, V, Cr, Mn, Fe, Co, Ni, Cu, Zn, As, Se, Mo, Ag, Cd, Sb, Ba, Tl, and Pb.

Amount [fg]	Blank	2.5	7.5	12	15	20
Mix	0	918	2787	4337	5474	7048
Si	0	459	1394	2169	2737	3524
K	0	9177	27874	43371	54737	70478

Table S7. Instrumental parameters for ICP-TOFMS measurements.

	ICP-TOFMS
Plasma Power [W]	1440
Sampling depth [mm]	3.5
Cone materials	Ni
Plasma gas flow [L min^{-1}]	14.0
Auxiliary gas flow [L min^{-1}]	0.80
Nebulizer gas flow [L min^{-1}]	0.90-1.0
Measurement mode	Collision cell technology (CCT)
CCT gas	93% He *(v/v)*, 7% H_2 *(v/v)*
Cell gas flow [mL min^{-1}]	4.20
Mass range	*m/z* =14-256

Table S8. The R^2 values for the nuclides that were used to create the library. The 10 elements that were below the threshold of $R^2<0.95$ are indicated in red and were not included in the library.

**R^2**		**R^2**		**R^2**	
0.999943	^{52}Cr	0.999692	^{56}Fe	0.938151	^{45}Sc
0.99991	^{111}Cd	0.999688	^{151}Eu	0.90227	^{39}K
0.99989	^{146}Nd	0.999685	^{175}Lu	0.866395	^{27}Al
0.999889	^{157}Gd	0.999678	^{153}Eu	0.413769	^{9}Be
0.999876	^{51}V	0.999678	^{238}U	0.281873	^{43}Ca
0.999862	^{114}Cd	0.999671	^{232}Th	0.075344	^{11}B
0.999839	^{59}Co	0.99966	^{169}Tm	-0.12515	^{23}Na
0.999827	^{60}Ni	0.999631	^{172}Yb	-0.31607	^{31}P
0.999818	^{89}Y	0.999614	^{66}Zn	-0.40267	^{24}Mg
0.999817	^{147}Sm	0.999522	^{75}As	-0.69136	^{7}Li
0.999814	^{88}Sr	0.999421	^{65}Cu		
0.999813	^{137}Ba	0.999374	^{115}In		
0.999813	^{163}Dy	0.999132	^{208}Pb		
0.999798	^{55}Mn	0.999024	^{205}Tl		
0.999791	^{139}La	0.998984	^{85}Rb		
0.999786	^{63}Cu	0.998938	^{53}Cr		
0.999782	^{71}Ga	0.998788	^{82}Se		
0.999765	^{166}Er	0.998385	^{78}Se		
0.999759	^{138}Ba	0.998168	^{209}Bi		
0.999744	^{185}Re	0.997175	^{44}Ca		
0.999732	^{140}Ce	0.997156	^{57}Fe		
0.999724	^{159}Tb	0.996852	^{133}Cs		
0.999724	^{141}Pr				
0.999703	^{165}Ho				
0.999696	^{69}Ga				

Table S9. The nuclides chosen from the set included in standards in the semiquantification approach, yielding the error calculation reported in the main text in Table 1.

Spleen Sample					Tumor Sample				
**Run 1**	**Run 2**	**Run 3**	**Run 4**	**Run 5**	**Run 1**	**Run 2**	**Run 3**	**Run 4**	**Run 5**
^{51}V	^{51}V	^{66}Zn	^{138}Ba	^{51}V	^{51}V	^{88}Sr	^{85}Rb	^{66}Zn	^{138}Ba
^{89}Y	^{53}Cr	^{71}Ga	^{139}La	^{66}Zn	^{89}Y	^{89}Y	^{89}Y	^{71}Ga	^{139}La
^{111}Cd	^{66}Zn	^{78}Se	^{140}Ce	^{71}Ga	^{111}Cd	^{133}Cs	^{133}Cs	^{78}Kr	^{141}Pr
^{114}Cd	^{75}As	^{88}Sr	^{141}Pr	^{88}Sr	^{114}Cd	^{147}Sm	^{138}Ba	^{78}Se	^{146}Nd
^{115}In	^{78}Se	^{133}Cs	^{146}Nd	^{89}Y	^{115}In	^{153}Eu	^{147}Sm	^{88}Sr	^{147}Sm
^{133}Cs	^{139}La	^{137}Ba	^{159}Tb	^{137}Ba	^{115}Sn	^{157}Gd	^{151}Eu	^{133}Cs	^{159}Tb
^{138}Ba	^{140}Ce	^{138}Ba	^{163}Dy	^{133}Cs	^{133}Cs	^{166}Er	^{157}Gd	^{138}Ba	^{163}Dy
^{139}La	^{147}Sm	^{139}La	^{172}Yb	^{140}Ce	^{138}Ba	^{169}Tm	^{163}Dy	^{146}Nd	^{172}Yb
^{140}Ce		^{146}Nd	^{176}Yb	^{141}Pr	^{139}La	^{172}Yb	^{165}Ho	^{153}Eu	^{175}Lu
^{147}Sm		^{153}Eu		^{147}Sm	^{147}Sm		^{172}Yb		

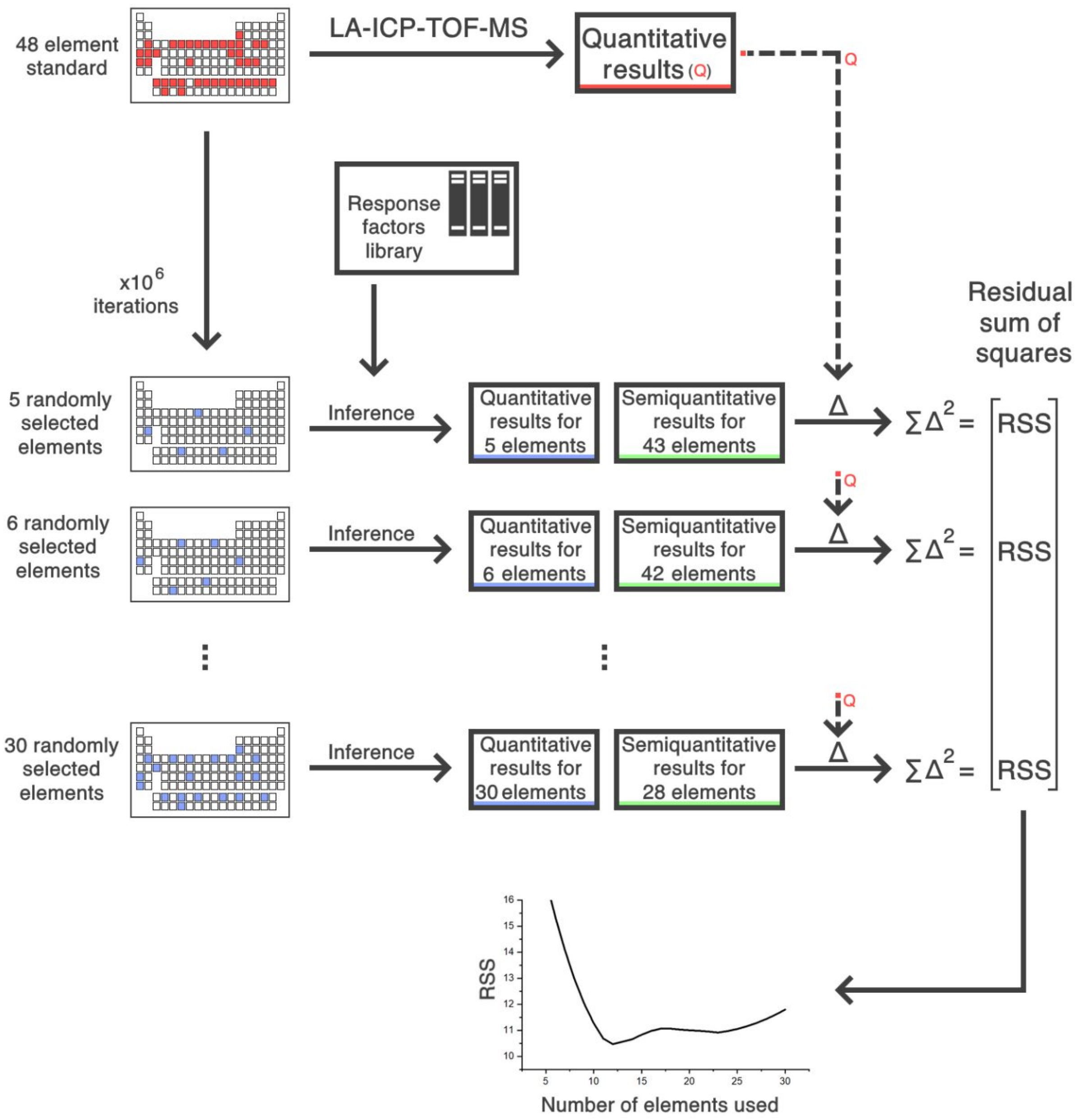

Figure S1. Overview on the workflow of the bootstrapping procedure used for the semiquantitative calculations.

Application functionality

1. The extraction of the calibration data from HDIP:

The h5 file obtained from TofPilot is processed via the "TOFWERK - icpTOF Batch Processor" module of HDIP to obtain signal intensity maps of the standards/sample. In the case of the gelatin micro-droplet standards an element that is present in gelatin is chosen to visualize the droplet standards. Recommended nuclides are for example $^{23}Na^+$ or $^{31}P^+$. Then, the "Labeled Segments" module is initiated and by using e.g. the "Paint" tool, each droplet can be marked as a segment, distinctly named and saved. Once segments for all standard droplets were created and saved, the "Inspect" tool is started. Here, all required parameters can be exported by clicking the "Export General Statistics For All Segments" and by selecting an appropriate folder for it to be saved in. The general statistics that are exported include the number of voxels and sum/integrated signal of the micro-droplet standards, which will be used for all further calculations. An example of the exported .xslx file is also uploaded, therefore users without HDIP can construct the file needed for SQ in the application described below. This file also serves as a template input file that the online app accepts (CalibrationTemplate.xlsx).

2. The online app (available at https://laicpms-apps.ki.si/webapps/home/) allows the user to perform a semiquantitative calibration using a template input file and calibrate raw input signal images.

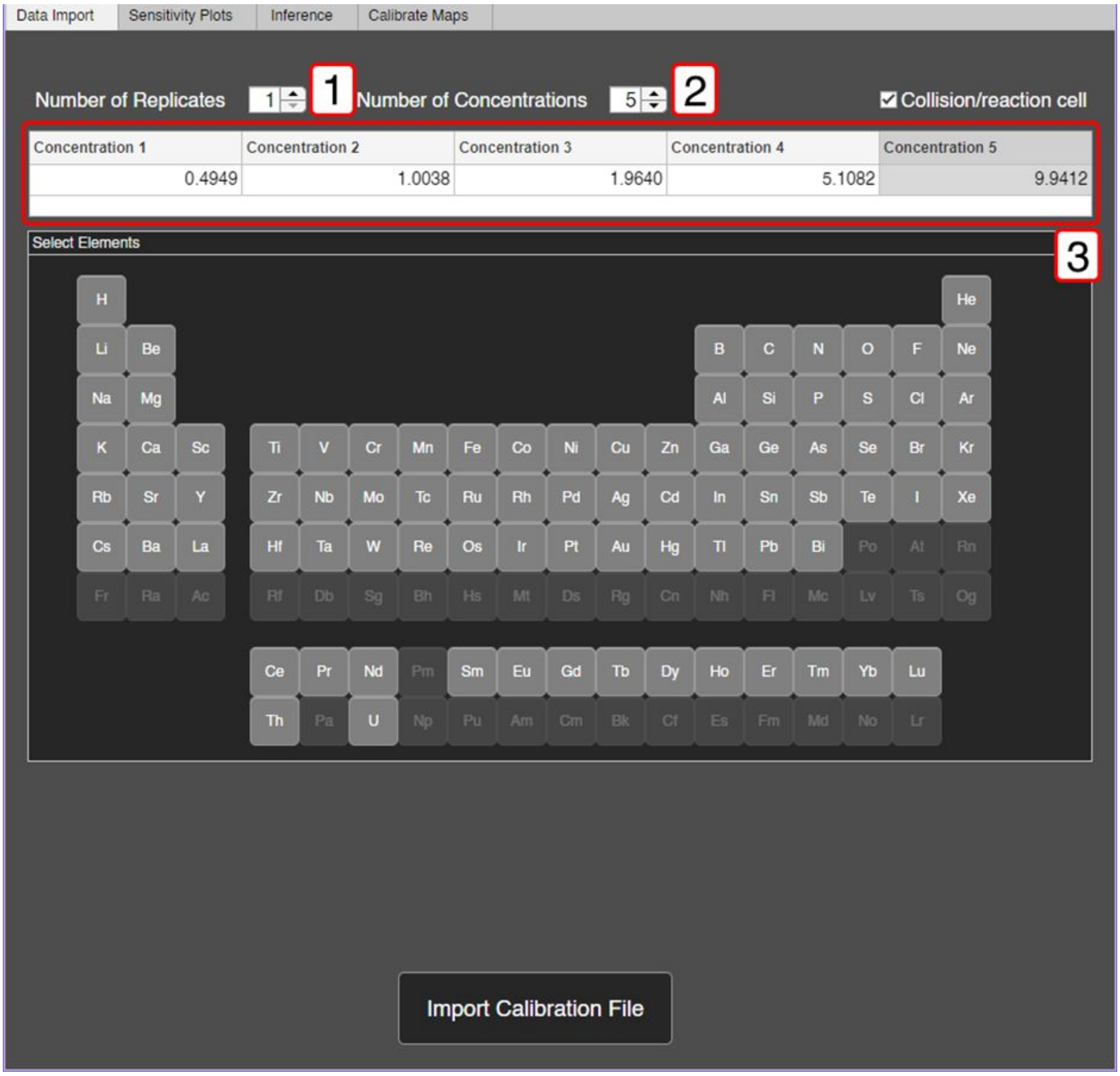

Figure S2. Application instructions 1.

After accessing, the user is presented with a number of inputs. The first set of inputs relates to the quantitative data used for semiquantitative calibration. After selecting the number of replicates (**1**) and the number of concentrations used (**2**), it is necessary to input all of the concentrations (in mg/L) by double clicking on the table fields (**3**). A template calibration file is provided (**CalibrationTemplate.xslx**) , which is compatible with the online app.

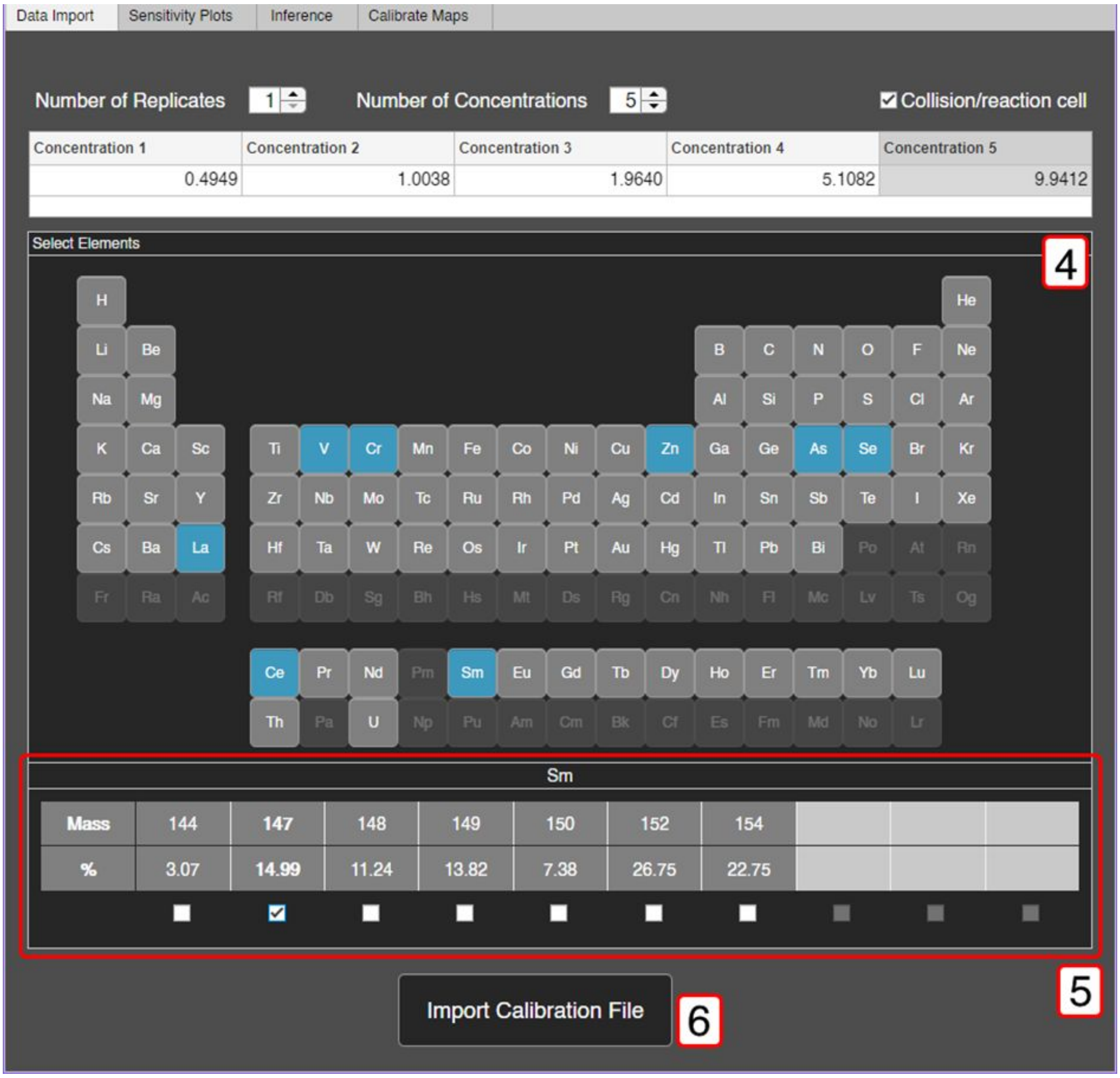

Figure S3. Application instructions 2.

After the calibration file details have been provided, the user then selects the elements (**4**) and masses (**5**) used for semiquantitative calibration. The optimal selection of elements is described in more detail in the article. Having selected the masses, the calibration file can be imported (**6**).

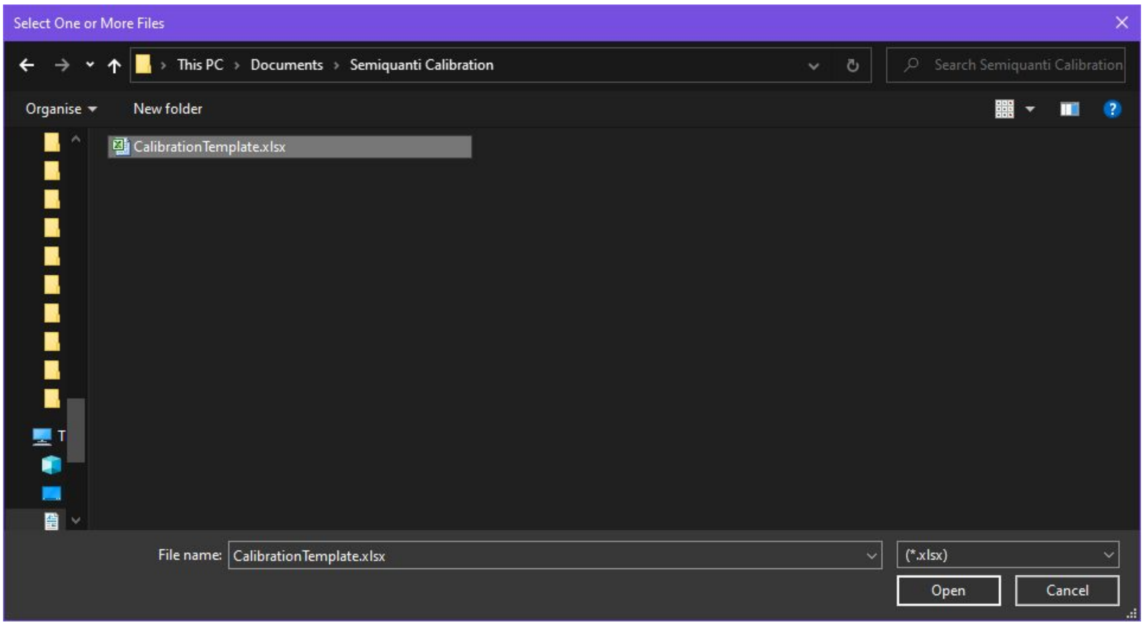

Figure S4. Application instructions 3.

Upon clicking the import button, a dialog box will appear. Here, the user selects the calibration file.

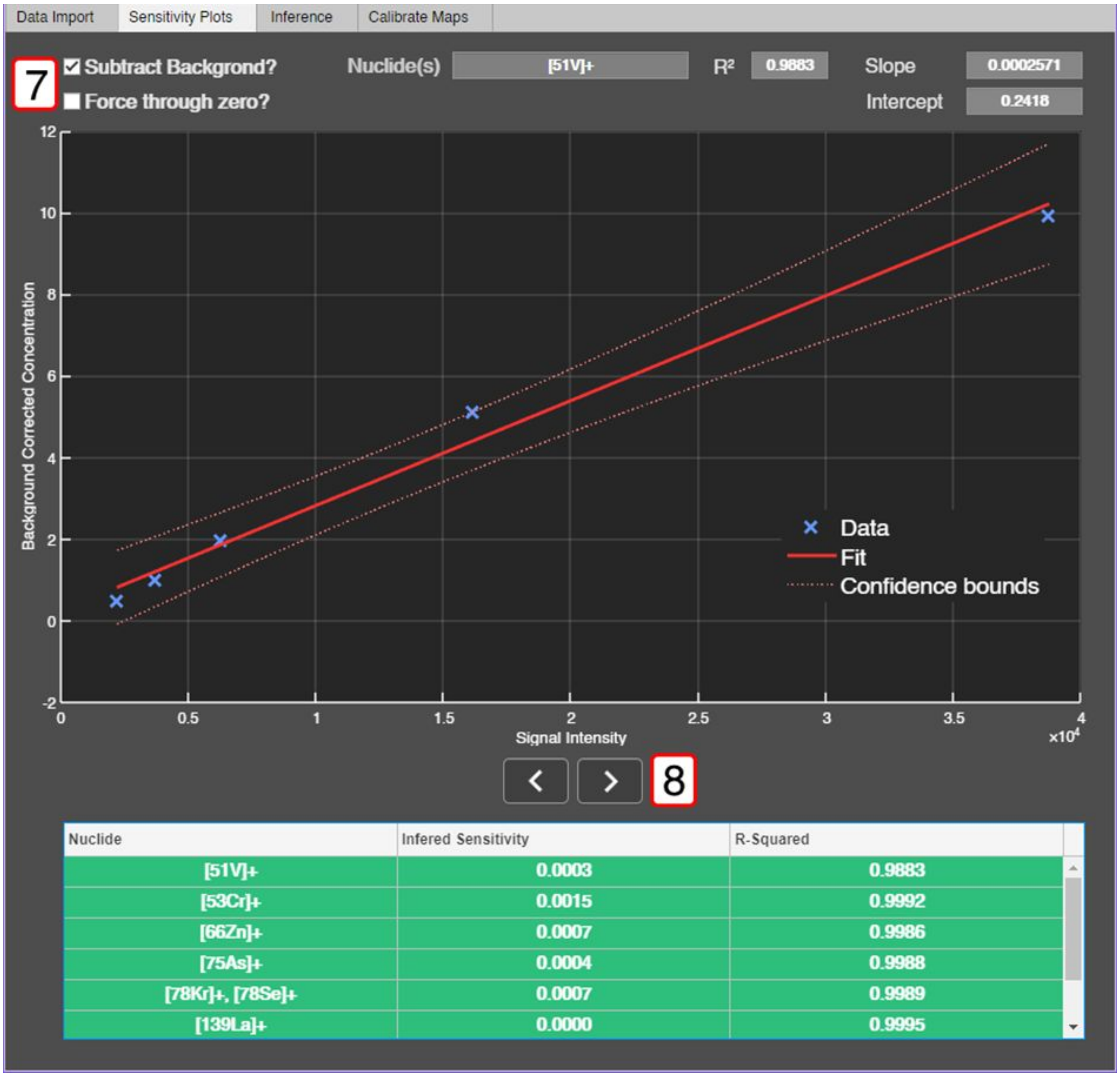

Figure S5. Application instructions 4.

The app will automatically conduct a linear regression from the input data and plot the results. Additional fit options (background subtraction and forcing through zero) are automatically re-calculated by clicking the check boxes (**7**). Two buttons allow for other nuclide fits to be displayed (**8**), all of which are listed in the table.

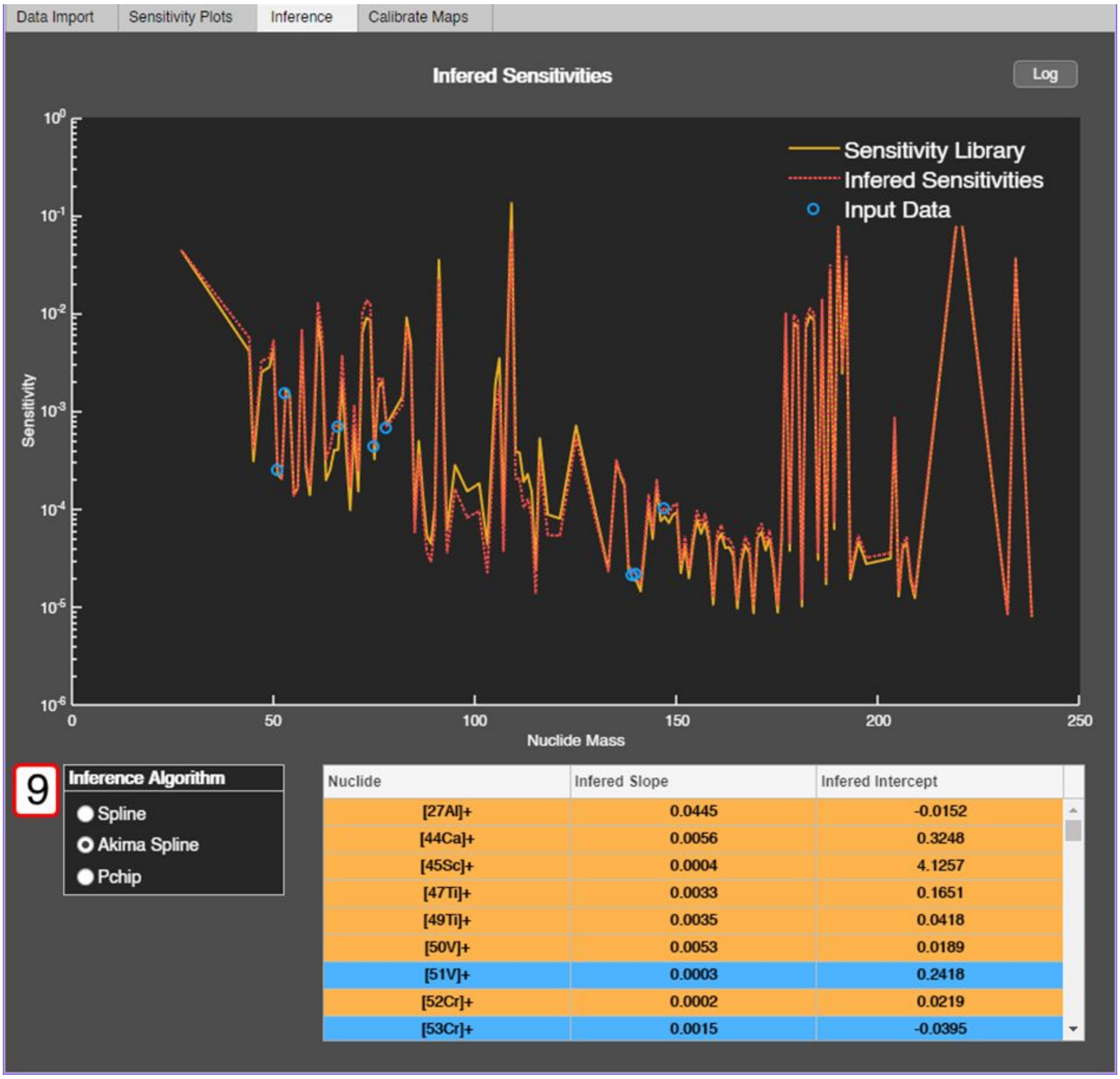

Figure S6. Application instructions 5.

Under the 'Inference' tab the user is presented with the inferred sensitivities for the whole mass range according to the empirically derived sensitivity library. Quantitatively derived sensitivities (from the input data) are marked in blue. Additionally, three options are provided for the fine tuning of the semiquantitative calibration (**9**).

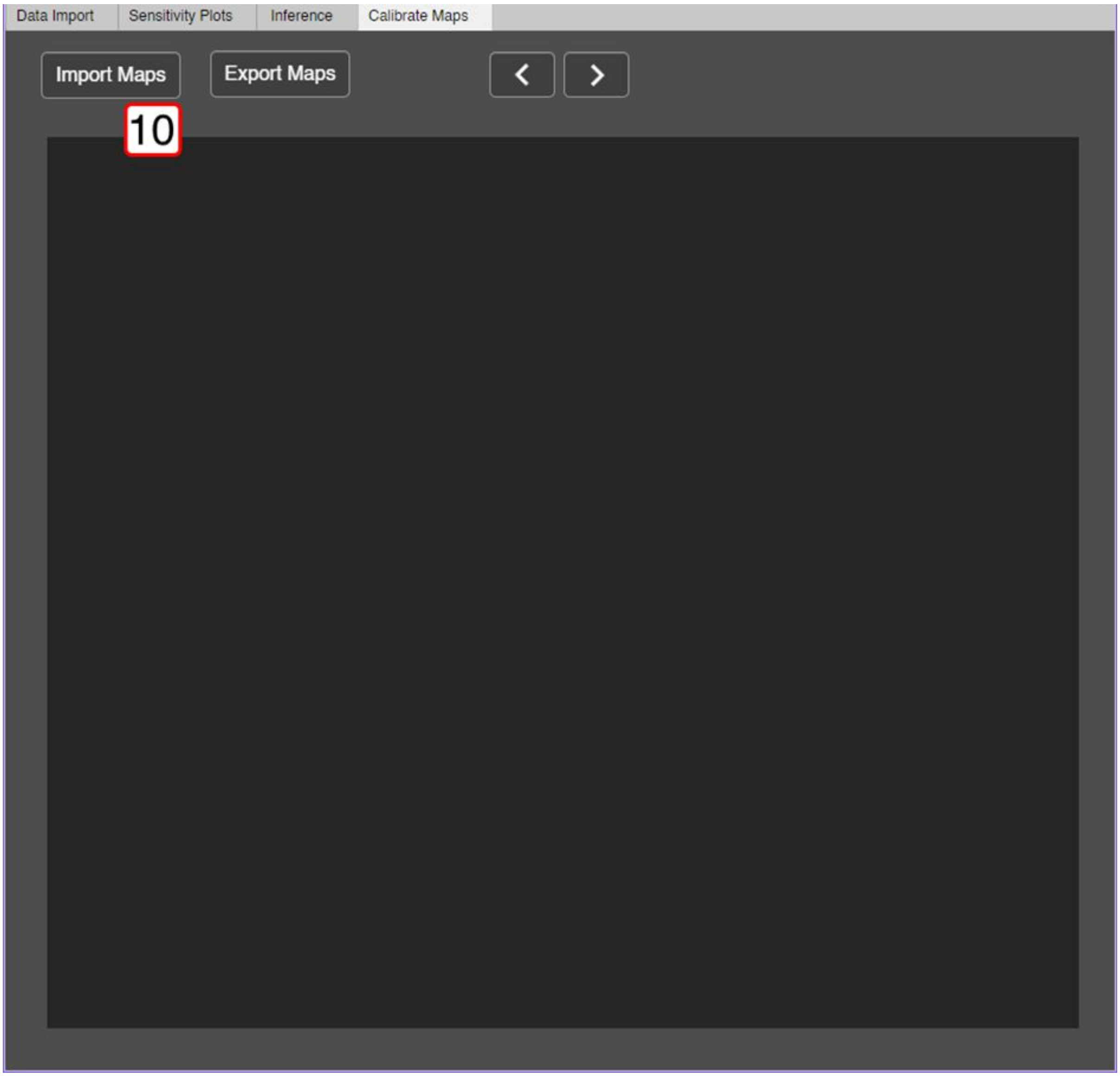

Figure S7. Application instructions 6.

The ‘Calibrate Maps’ tab offers the ability to bulk import .csv maps (in counts) and calibrate them semiquantitatively or quantitatively (where available). This is done by clicking on the ‘Import Maps’ button (**10**).

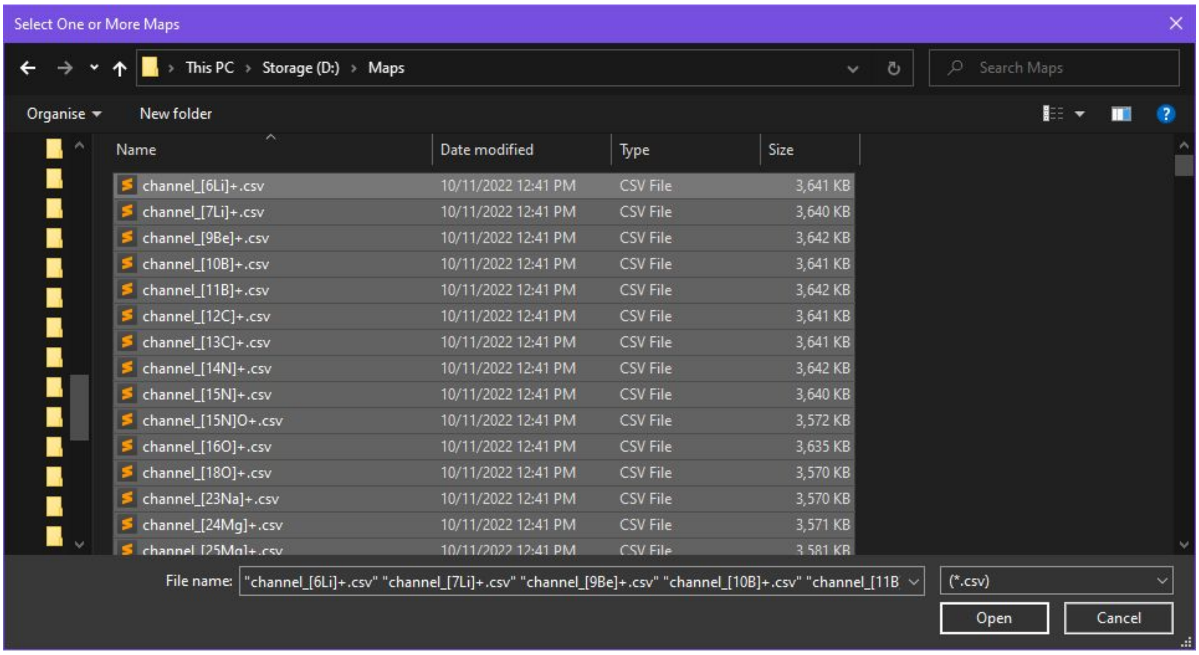

Figure S8. Application instructions 7.

The user selects one or more maps to calibrate. The app automatically parses the name of the .csv files for the nuclide name. The maps should be labeled in the following format:

[nA]+, where **n** is the nuclide mass and the **A** is the label (for example: **[24Mg]**+, or **[238U]**+).

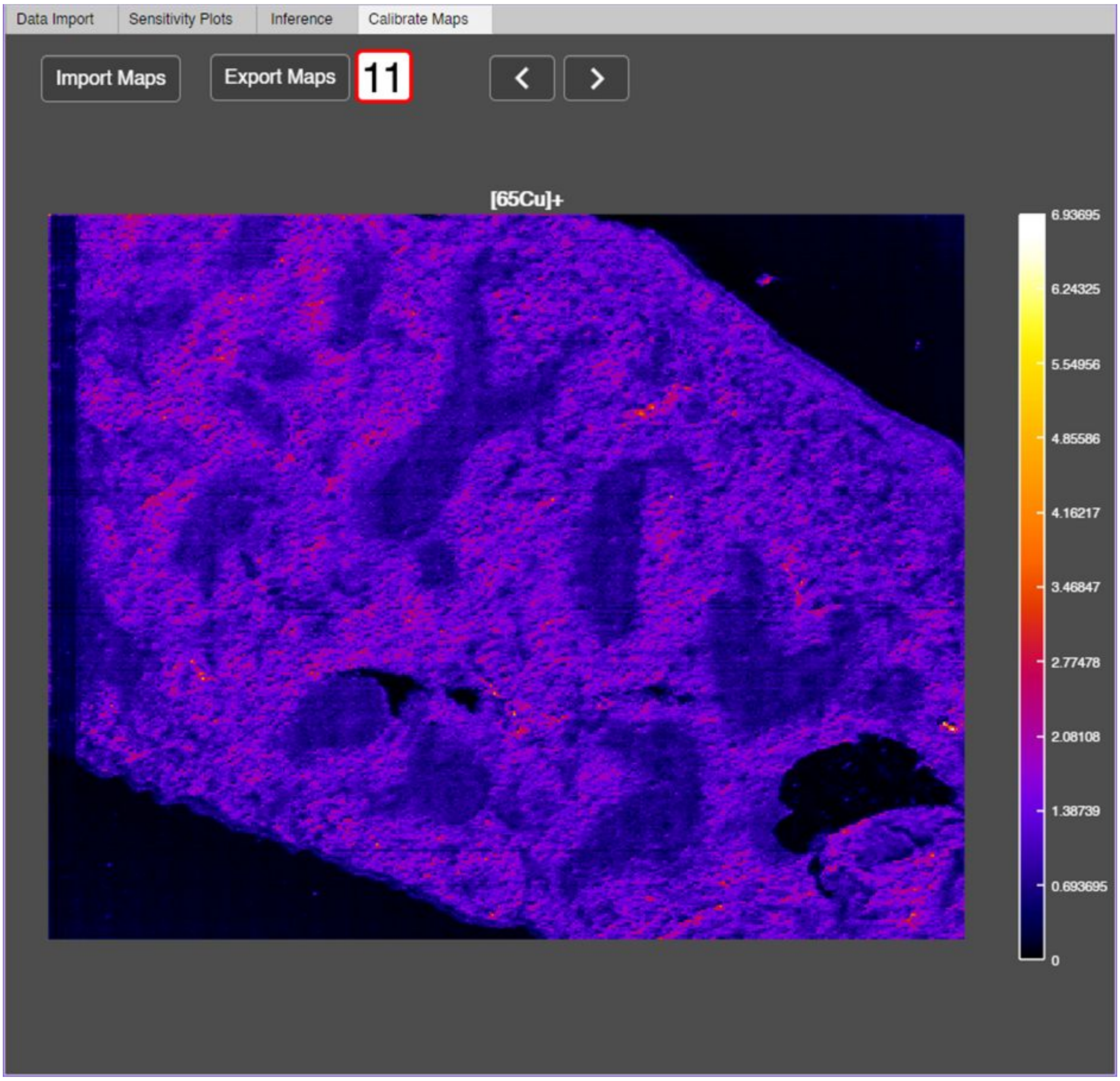

Figure S9. Application instructions 8.

After the maps have been imported and calibrated, they can be exported and saved to a desired location (**11**).
